# Acoustic Manipulation of Droplets under Reduced Gravity

**DOI:** 10.1038/s41598-019-53281-4

**Published:** 2019-11-12

**Authors:** Koji Hasegawa, Ayumu Watanabe, Yutaka Abe

**Affiliations:** 1 Department of Mechanical Engineering, Kogakuin University, Tokyo, Japan; 2 Graduate School of Systems and Information Engineering, University of Tsukuba, Tsukuba, Japan; 3 Faculty of Engineering, Information and Systems, University of Tsukuba, Tsukuba, Japan

**Keywords:** Fluid dynamics, Acoustics

## Abstract

Contactless manipulation of matter is essential for studying physical phenomena. Acoustic manipulation of liquid samples using ultrasonic phased arrays provides a novel and attractive solution for mid-air manipulation, such as levitation, transportation, coalescence, mixing, separation, evaporation, and extraction, with a simple and single sequence. Despite the importance of gravity in droplet dynamics, its effect on a levitated droplet with an ultrasonic phased array remains unclear. To disseminate acoustic manipulation, better understanding of the fundamental physics of a droplet manipulated by ultrasonic phased arrays is required. Here, we show contactless levitation, transportation, and coalescence of multiple droplets under both ground and reduced gravity. Under ground gravity, the possible levitation size of the sample is limited to below the half wavelength of sound. Under reduced gravity, however, droplets that are larger than the limit can be successfully levitated, transported, and coalesced. Furthermore, the threshold of sound pressure for droplet levitation and manipulation could be minimised with the suppression of nonlinear acoustic phenomena under reduced gravity. These insights promote the development of contactless manipulation techniques of droplets for future space experiment and inhabitancy.

## Introduction

The acoustic levitation method (ALM) of sample manipulation^1–6^ is of great importance and a promising candidate for potential mid-air lab-on-a-drop applications, including the solidification of materials^7^, chemical analysis^8,9^, X-ray crystallography^10^, DNA transfection^11^, blood analysis^12^, and microreactors^13^. ALM has been studied for the contactless handling of matter in air. Because it enables us to achieve container-free processing, ALM can prevent heterogeneous nucleation and contamination from a container wall^2^. Foresti *et al*.^14–16^ investigated the transport and coalescence of acoustically levitated droplets with multiple ultrasonic transducers and reflectors. Marzo *et al*.^3^ demonstrated an innovative technique for particle and droplet manipulation by forming arbitrary sound potential fields with appropriate phase differences using an ultrasonic phased array. Although ALM enables highly precise manipulation using sound fields irrespective of sample properties^1^, nonlinear and unsteady phenomena (e.g., interfacial instability and atomisation^15^ and acoustic streaming^17,18^) emerge in droplet manipulation. Thus, the characteristics of single acoustic levitator were investigated in the last decades^19–27^. However, the dynamics of droplet manipulation via ultrasonic phased arrays have not been fully investigated.

To the best of our knowledge, ALM via ultrasonic phased arrays has not been applied under different gravity levels^28^. Although a droplet can be levitated under the condition of earth’s gravity, a strong acoustic energy is required to overcome it. This strong energy causes the above-mentioned non-linear and dynamic behaviours. In contrast, because the required acoustic energy is much smaller in the lower gravity environment, it is an ideal environment that can minimize the complicated phenomena and enable us to reveal the underlying nature of droplet dynamics. It is of necessity and importance to demonstrate the feasibility study of droplet manipulation under lower gravity to obtain physical insights for the further development of ALM.

This study aims to investigate and better understand contactless liquid manipulation under both ground and reduced gravity. Experiments were conducted under the condition of an airplane in parabolic flight to achieve the following processes: coalescence, mixing, separation, and evaporation. In this paper, we demonstrate a feasibility study and fundamental physics for the levitation, transportation, and coalescence of droplets using an ultrasonic phased array under varying gravity. Our insights could pave the way towards droplet manipulation for lab-in-a-drop applications both on Earth and in space.

## Results

### Experiments under ground gravity

**Figure 1:**
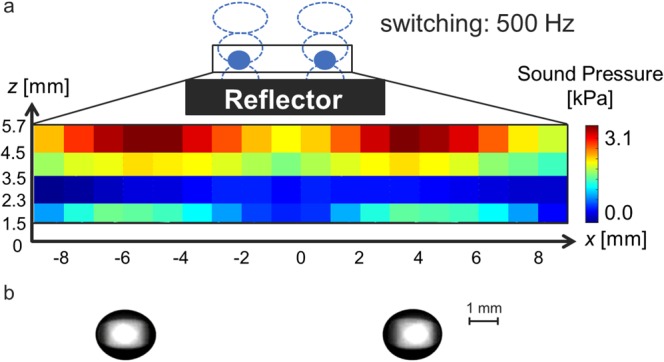
Sound pressure distribution and droplet levitation on ground. (**a**) Sound pressure distribution in the *x*-*z* plane. (**b**) Snapshot of levitation of a pair of water droplets: *d = *2.1 mm (left) and *d = *1.9 mm (right).

Prior to the feasibility study under reduced gravity, we conducted preliminary experiments of droplet levitation, transportation, and coalescence under ground gravity. Figure 1a represents the distribution of RMS sound pressure with two focal points. The distance between the focal points was set to 10 mm. The loop and node were formed at both focal points, and then the formation of a pair of acoustic standing waves were confirmed by switching the focal points. In this study, ethanol droplets were used for the experiments. Figure 1b presents a snapshot of the levitation behaviour of a pair of ethanol droplets. The pair of millimetre-sized ethanol droplets were simultaneously and stably levitated at each standing wave.

**Figure 2:**
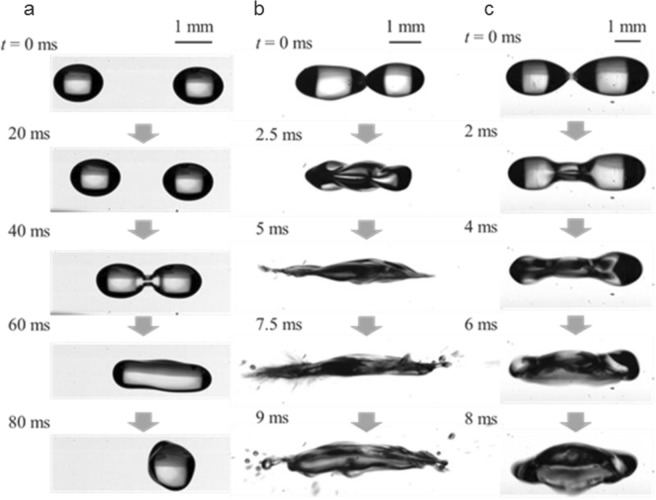
Patterns of ethanol droplet manipulation under ground gravity. (**a**) Successful droplet coalescence. Equivalent diameters of droplets *d* and applied RMS sound pressure *p* were 1.8 mm (left), 1.8 mm (right), 2.2 mm after the droplets coalesced, and 1.2 kPa. (**b**) Droplet atomisation immediately after coalescence: *d = *1.9 mm (left), *d = *1.7 mm (right), and *p* = 1.8 kPa. (**c**) Droplet falling immediately after coalescence: *d = *2.1 mm (left), *d = *2.2 mm (right), and *p = *1.7 kPa. See Supplementary Videos 1–3 as well.

Figure 2 shows the typical pattern of droplet levitation under ground gravity. The droplets can be stably levitated, transported, and coalesced with relatively small droplets and RMS sound pressure (Fig. 2a and Supplementary Video 1) can be implemented on them. With larger sound pressure and droplets, however, the droplets get atomised (Fig. 2b and Supplementary Video 2) or fall (Fig. 2c and Supplementary Video 3) immediately after droplet coalescence. Droplet atomisation can be attributed to the interfacial instability^29^ on the droplet surface with higher sound pressure. In the case when the droplets fall, larger droplets could not counteract the body force on the droplets under gravity. For stable levitation and manipulation of the droplets, the acoustic radiation force should exceed the gravitational force on the droplet ($\geqq$*ρVg*) while below the surface force to maintain the droplet interface. Here *ρ, V*, and *g* are the density and volume of the droplet, and gravitational acceleration, respectively. Precise tuning of droplet size and sound pressure was required under ground gravity.

### Experiments under reduced gravity

**Figure 3:**
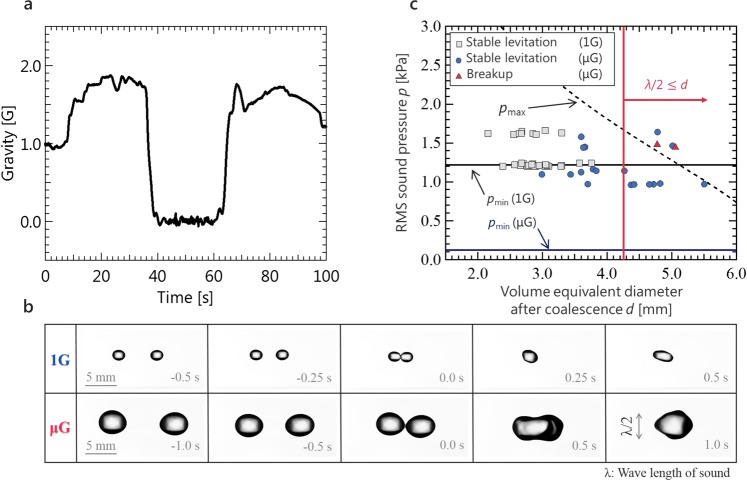
Acoustic manipulation under reduced gravity environment. (**a**) Time history of vertical gravity during parabolic flight. (**b**) Comparison of manipulated water droplets between ground gravity and reduced gravity. 1 G and μG denote the ground and reduced gravity, respectively. Equivalent diameters of droplets and applied RMS sound pressure were 2.0 mm (1 G, left), 2.0 mm (1 G, right), 1.7 kPa (1 G), 4.4 mm (μG, left), 4.4 mm (μG, right), and 0.9 kPa (μG). See Supplementary Video 4 (top: 1 G, bottom: μG) as well. (**c**) Comparison of stable levitation condition after coalescence between the ground and reduced gravity conditions. The dashed and solid lines denote the upper and lower (1 G and μG) limits of levitation predicted by Eqs (1) and (2).

Experiments in reduced gravity were conducted via parabolic flights using an aircraft (Gulfstream-II, Diamond Air Service Inc., Japan). Figure 3a shows an example of the gravitational acceleration profile during the parabolic flights. Here, 1 G is 9.81 m/s^2^. After the first 30 s, the gravitational acceleration level reached 10^−2^ G during 20 s. After the confirmation of formation of the standing wave, two water droplets were injected from a syringe under the reduced gravity condition. The behaviour of the levitated droplet was observed using a high-speed video camera and the results were compared to those of the ground gravity experiment. Figure 3b and Supplementary Video 4 show the comparison results of the levitation and coalescence behaviours of the droplets between the reduced and ground gravity conditions. In the reduced gravity condition, the levitation, transport, and coalescence of large droplets (i.e., larger than the half wavelength of sound) can be successfully performed. Accordingly, we achieved the levitation, transport, and coalescence of acoustically levitated droplets in the reduced gravity conditions. The largest difference between the ground and reduced gravity conditions was observed in the maximum size of droplets and minimum value of sound pressure for manipulating the droplets. Figure 3c shows the evaluation results of the stable levitation condition after coalescence by experimentally determining the relationship between the droplet diameter after coalescence and sound pressure. The black solid line and blue broken line denote the lower limit of sound pressure to maintain the suspended droplet under ground gravity and reduced gravity, respectively, which can be described as Eq. (1)^30^:

1 $${p}_{{\rm{\min }}}=\sqrt{\frac{1.6{\rho }_{L}{\rho }_{G}\,g{c}^{2}}{k}},$$

where, *ρ *is the density, subscripts L and G represent the liquid phase and gas phase, respectively, *g* is the gravitational acceleration, *c* is the speed of sound, and *k* is the wave number. For the reduced gravity experiment, the lower limit of sound pressure was calculated using *g* = 0.01 G in Fig. 3c. The broken curve represents the upper limit and is calculated as follows^31^:

2 $${p}_{{\rm{\max }}}=\sqrt{\sigma {\rho }_{G}{c}^{2}(\frac{3.2}{d}-\frac{1.3\pi }{\lambda })},$$

where *σ* is the surface tension, *d* is the diameter of the droplet, and *λ* denotes the wavelength. In the case of ground gravity, the droplet was considered to stably coalesce under the condition between the theoretical lower and upper limits. In the case of reduced gravity, the droplet was found to stably coalesce at sound pressures below the lower limit of the case of ground gravity. This result suggests that the sound pressure required for droplet manipulation can be minimised and the suppression of nonlinear acoustic phenomena was accordingly considered in the reduced gravity experiment. Above the higher limit condition, we demonstrated that the droplet successfully levitated but atomized in some cases. One possible reason for this may be the sensitive coalescence dynamics and complex interfacial instability of the droplets; however, the exact reason remains unclear because when the droplets merge, unsteady interfacial behaviors can be developed (Fig. 2b and Supplementary Video 2). This sensitive behavior is most likely to affect droplet coalescence by generating fluctuations of unsteady flow and pressure fields between the approaching droplets. As a future work, we intend to investigate the instantaneous interfacial motions of droplets and interactions among attracting droplets immediately before coalescence for more stable manipulation.

## Discussion

**Figure 4:**
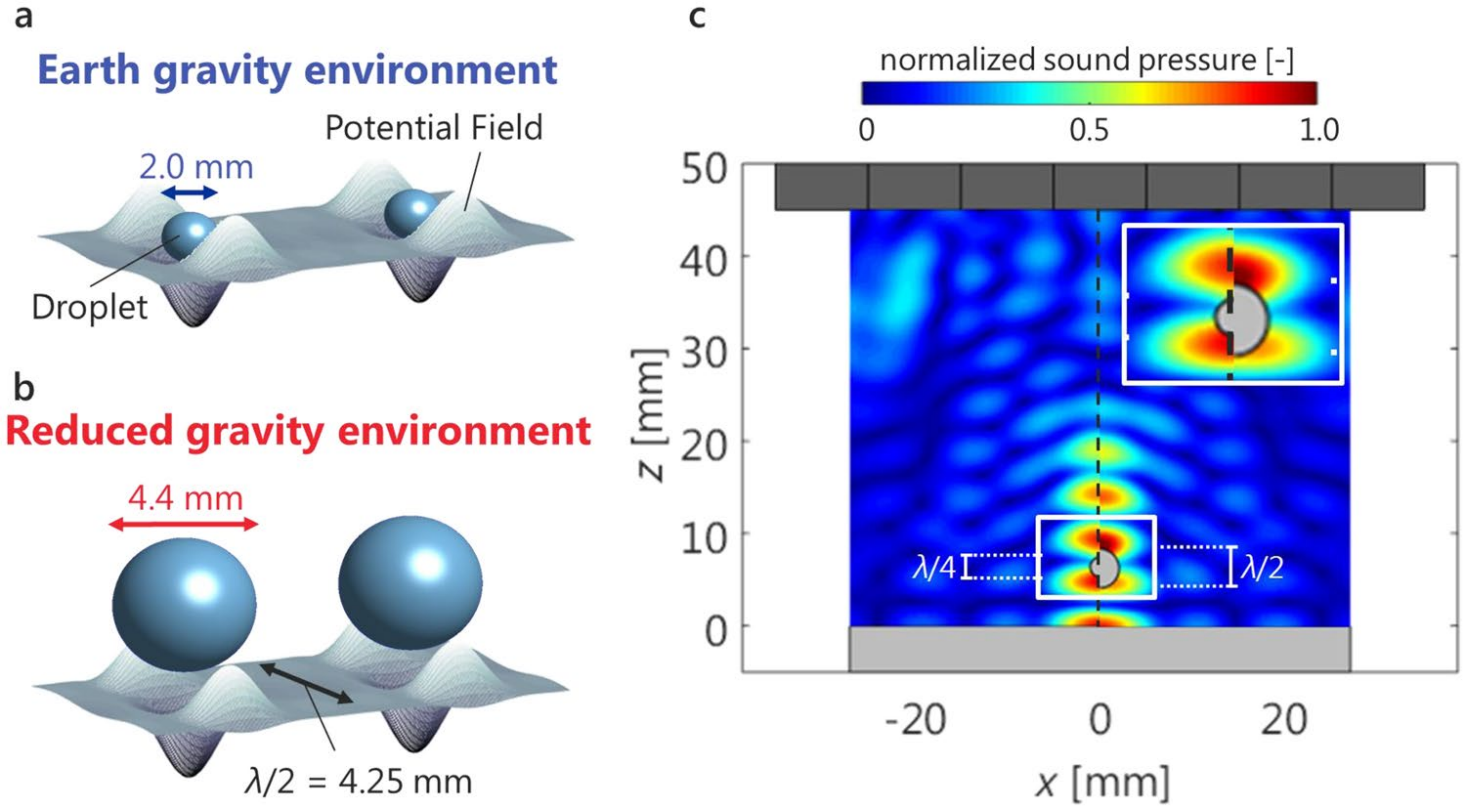
Droplet levitation beyond the limit under reduced gravity. A schematic diagram showing a comparison of scale between the acoustic potential and levitated droplet under (**a**) ground gravity and (**b**) reduced gravity. (**c**) Normalised sound pressure distribution with a solid sphere of *λ*/4 (left) and *λ*/2 (right). The inset is close-up image near the solid sphere. Sound pressure is normalised using the maximum sound pressure in the distribution.

Through the reduced gravity experiment, stable coalescence of the droplet at sound pressures below the lower limit of under ground gravity can be verified. As shown in Fig. 4, in acoustic levitation using an acoustic standing wave, the sample levitated by tapping at the potential well and the interval of the potential well corresponds to the half wavelength of the sound. Although the theoretical and experimental lower limits of stable levitation under reduced gravity (*p*_min_(μG) in Fig. 3c) can be expanded, the allowable diameter of the sample has been considered to be limited to the half wavelength or less (Fig. 4a). However, the experimental results show that the droplets that are larger than the half wavelength (i.e., larger than 4.25 mm) can be levitated, transported, and coalesced under the reduced gravity environment. To obtain clear insights into the force acting on the levitated sample, we numerically calculated the acoustic potential^32^ and sound pressure in the vicinity of the levitated solid sphere^33^ under reduced gravity using the distributed point source method (DPSM)^34^. For *d* = *λ*/4 (in Fig. 4c, left), it is understood that the acoustic radiation pressure acting on the lower surface of the sphere exceeds the value of the spherical upper surface. Therefore, the vertical retention force against gravity enables the solid sphere to suspend in air. For *d* = *λ*/ 2 (right), the acoustic radiation pressure of the upper surface is higher than that of the lower surface. This indicates that a holding force in the vertical upward direction is not sufficient to counteract the gravity. In this case, the solid sphere is considered to fall under ground gravity. From this result, there is a condition under which an appropriate radiation pressure acts at a certain height, and by setting the reduced gravity environment, the droplet spontaneously moves to a position that satisfies the condition. Accordingly, it is inferred that the droplets larger than the half wavelength of 4.25 mm (in Fig. 3b) can be suspended.

These experimental results demonstrate the applicability of the potential technology for the further development of contactless manipulation techniques for liquid samples. Additionally, in the reduced gravity environment, the droplets with diameters exceeding the half wavelength of sound can be acoustically manipulated, which was previously considered impossible^35–37^. This enables sample manipulation over a wider range of droplet sizes. The results show that acoustic manipulation using ultrasonic phased array provides a novel solution for noncontact mid-air sample manipulation^38^ under varying gravity. Our demonstration provides the feasibility of contactless levitation, transportation, and coalescence of droplets as a first step in fluid manipulation for potential applications.

## Methods

### Experimental setup

**Figure 5:**
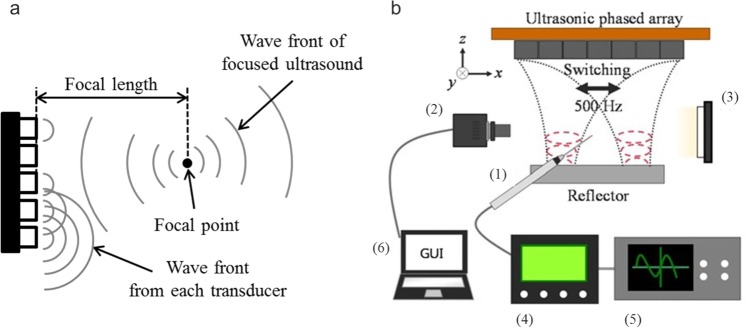
Schematic diagram of experimental apparatus. (**a**) Concept of focused ultrasound transmitted from an ultrasonic transducer array. (**b**) Schematic of experimental setup: (1) probe microphone, (2) high-speed video camera). (3) LED light, (4) amplifier, (5) oscilloscope, and (6) computer.

Acoustic levitation using an ultrasonic phased array was implemented in this study. By generating sound waves with a controlled phase, the focal point of sound is formed at an arbitrary position. A localised standing wave can then be generated near the focal point by reflecting the focused ultrasound using a reflector. We used a 7 × 7 square transducer array consisting of 49 small ultrasonic transducers. The diameter and frequency of the transducer were 10 mm and 40 kHz, respectively. Phase control of the sound transmitted from each transducer is required to generate an ultrasonic focal point. We realised this control using a field programmable gate array (FPGA) (Altera Co., Cyclone-IV DE0-Nano). The experimental apparatus is shown in Fig. 5. The focal length and the distance from the transducer to the reflector were 45 mm each. Two droplets were successfully levitated by switching two focal points at the frequency of 500 Hz. We therefore selected the switching frequency to be 500 Hz^5^. The ambient temperature was 25 °C for both the ground and reduced gravity experiments. The relative humidity was approximately 60% and 10% for the ground and reduced gravity, respectively. However, despite the difference between the ground and reduced gravity conditions, both experiments were conducted within a sufficiently short time (~20 s), as compared to the characteristic time of the evaporation of a water droplet (*d*^2^/*D*~*O*(10^3^) s, *D* (~10^−9^ m^2^/s) is the diffusion coefficient of water^39^). The RMS sound pressure was tuned between 0.9 and 1.8 kPa to ensure the stable levitation of the droplet. To quantitatively evaluate the sound field, the sound pressure was measured using a probe microphone (Bryel & Kjaer, Type 4138, diameter: 1/8 inch). The microphone was fixed on the traverse device, which could move along the *x*, *y*, and *z* directions. The behaviour of the levitated droplets was observed from the side using a high-speed video camera (Photron Co., Ltd. FASTCAM-Mini UX100) with back-light illumination. Table 1 lists the test fluids and their physical properties^40^.

**Table 1:** Properties of fluids.

Sample	Density *ρ*_*L*_ (kg/m^3^)	Surface tension σ (mN/m)
Water	998	72.7
Ethanol	784	21.5

**Figure 6:**
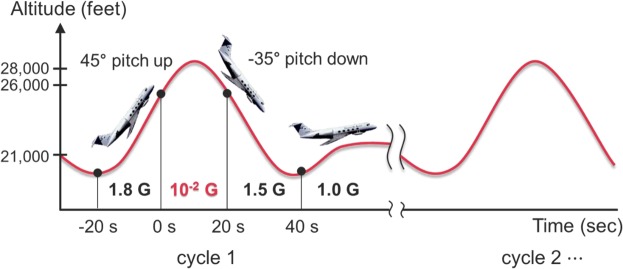
Schematic diagram of parabolic flight.

Experiments under reduced gravity were conducted via aircraft parabolic flights (Gulfstream-II, Diamond Air Service Inc., Japan), as shown in Fig. 6. We conducted the experiments within 20 s of 10^−2^ g.

### Calculation method of sound field

DPSM^24,25^ was used to model the sound field without computational mesh by distributing the point sources of sound at the boundary. Emitters and reflectors were discretised by point sources to represent the sound field. The sound pressure *p*_m_ and velocity *v*_m_ at a distance *r*_mn_ generated by the sound wave radiated from the point source m are given by

3 $${p}_{{\rm{m}}}({r}_{{\rm{mn}}})={A}_{{\rm{m}}}\frac{\exp \,i(k{r}_{{\rm{mn}}}-\omega {t}_{{\rm{m}}})}{{r}_{{\rm{mn}}}}={A}_{{\rm{m}}}G({r}_{{\rm{mn}}}),$$

4 $${v}_{{\rm{m}}}({r}_{{\rm{mn}}})=\frac{{\boldsymbol{n}}\cdot {{\boldsymbol{r}}}_{{\rm{mn}}}}{i\omega {\rho }_{G}}\frac{\partial p}{\partial r}={A}_{{\rm{m}}}M({r}_{{\rm{mn}}}),$$

where *A*_m_ is the strength of the *m*^th^ point, *k = *2π/*λ* is a wave number, *ω* is the angular frequency, and *ρ*_G_ is the density of air. For the ultrasonic phased array, sound waves radiated from each transducer were excited after different time intervals *Δt*_m_ to focus the sound waves. Then, the term *t*_*m*_ in Eq. (3) was expressed in terms of phase difference from a reference time *t*:

5 $${t}_{{\rm{m}}}=t-\varDelta {t}_{{\rm{m}}}.$$

If there are N point sources, the total value at point *x* is given by

6 $$p(x)=\mathop{\sum }\limits_{{\rm{m}}=1}^{N}{p}_{{\rm{m}}}({r}_{{\rm{mn}}}),$$

7 $$v(x)=\mathop{\sum }\limits_{{\rm{m}}=1}^{N}{v}_{{\rm{m}}}({r}_{{\rm{mn}}}).$$

If there are secondary sound sources such as a reflector, the amplitudes of the sound sources are determined to satisfy the boundary condition. *N* point sources, for which the amplitude is unknown, are distributed at both primary (e.g., transducers) and secondary sound sources (e.g., reflector). Here, the sound sources are assumed to be a sphere of radius *r*_s_ and the radiation points are assumed to be located at a position retracted by *r*_s_ from the boundary. Considering the velocity *V*_0_ for the primary sound sources and velocity 0 for the secondary sources, the following equation can be derived:

8 $$\{{v}_{{\rm{m}}}\}={}^{t}\{{V}_{0},\cdots \,{V}_{0},\,0,\cdots \,0\},$$

9 $$\{{A}_{{\rm{m}}}\}={[{M}_{{\rm{mn}}}]}^{-1}\{{v}_{{\rm{m}}}\}.$$

Because *N* equations were given for *N* unknown sound sources, the amplitudes of the unknown sound sources were determined.

### Statistical analysis

The uncertainty in droplet diameter is <2% because when *d* = 1.7 mm (the smallest case in the present study), the standard deviation with three measurements was less than 2 pixels, with a spatial resolution of ~15 μm/pixel. The uncertainty in sound pressure is <6% because when *p* = 0.9 kPa under reduced gravity, the standard deviation with three measurements was less than 0.05 kPa.

## Supplementary information

Supplementary Video 1

Supplementary Video 2

Supplementary Video 3

Supplementary Video 4

## Data Availability

The datasets generated during and/or analysed during the current study are available from the corresponding author on reasonable request.
